# Seasonal variation of diarrhoeal pathogens among Guinea-Bissauan children under five years of age

**DOI:** 10.1371/journal.pntd.0011179

**Published:** 2023-03-13

**Authors:** Sointu Mero, Tinja Lääveri, Johan Ursing, Lars Rombo, Poul-Erik Kofoed, Anu Kantele

**Affiliations:** 1 Human Microbiome Research Program, Faculty of Medicine, University of Helsinki, Helsinki, Finland; 2 Meilahti Infectious Diseases and Vaccine Research Center, MeiVac, Department of Infectious Diseases, University of Helsinki and Helsinki University Hospital, Helsinki, Finland; 3 Department of Computer Science, Aalto University, Espoo, Finland; 4 Department of Infectious Diseases, Danderyds Hospital, Stockholm, Sweden; 5 Department of Clinical Science, Karolinska Institutet, Danderyd Hospital, Stockholm, Sweden; 6 Bandim Health Project, Indepth Network, Bissau, Guinea-Bissau; 7 Department of Medical Biochemistry and Microbiology, Uppsala University, Uppsala, Sweden; 8 Centre for Clinical Research, Sörmland County Council, Eskilstuna, Sweden and Uppsala University, Uppsala, Sweden; 9 Department of Paediatrics and Adolescent Medicine, Lillebaelt Hospital, University Hospital of Southern Denmark, Kolding, Denmark; University of Virginia School of Medicine, UNITED STATES

## Abstract

**Background:**

Diarrhoea remains a major cause of childhood morbidity and mortality in low-income countries (LICs). The frequency of diarrhoeal episodes may vary by season, yet few prospective cohort studies have examined seasonal variation among various diarrhoeal pathogens using multiplex qPCR to analyse bacterial, viral and parasitic pathogens.

**Methods:**

We combined our recent qPCR data of diarrhoeal pathogens (nine bacterial, five viral and four parasitic) among Guinea-Bissauan children under five years old with individual background data, dividing by season. The associations of season (dry winter and rainy summer) and the various pathogens were explored among infants (0–11 months) and young children (12–59 months) and those with and without diarrhoea.

**Results:**

Many bacterial pathogens, especially EAEC, ETEC and *Campylobacter*, and parasitic *Cryptosporidium*, prevailed in the rainy season, whereas many viruses, particularly the adenovirus, astrovirus and rotavirus proved common in the dry season. Noroviruses were found constantly throughout the year. Seasonal variation was observed in both age groups.

**Conclusion:**

In childhood diarrhoea in a West African LIC, seasonal variation appears to favour EAEC, ETEC, and *Cryptosporidium* in the rainy and viral pathogens in the dry season.

## Author summary

Childhood diarrhoea, a major cause of mortality in low-income countries (LICs), is known to be influenced by seasonal variations. Studies on the seasonality of diarrhoeal pathogens have mostly been conducted in the temperate zone where the seasons differ by temperature. In Guinea-Bissau and many other LICs, seasonality is characterized by precipitation changes with negligible temperature variations. The monsoon-type rainy season lasts from June to October and the dry season from November to May. We expect this seasonality to influence stool pathogen distribution.

To investigate the associations between various diarrhoeal pathogens and seasonality in childhood diarrhoea in Guinea-Bissau, we explored a large variety of bacterial, viral and parasitic agents among children with and without diarrhoea during the rainy versus dry seasons.

We found diarrhoeal pathogens not to be equally present over the year but show seasonal variation with respect to age and precipitation. The waterborne *Cryptosporidium* showed highest prevalence during the wetter months, while viral pathogens (adenovirus, astrovirus and rotavirus) were found most frequently during the arid months. Of bacterial pathogens, EAEC, ETEC, and *Campylobacter* prevailed in the rainy season. Such data add to our understanding of childhood diarrhoea in LICs and serve as a tool for devising preventive measures.

## Introduction

The seasonality of diarrhoeal pathogens has mostly been examined in temperate regions [1], where seasons are mainly characterized by variations in temperature. Fewer studies have been conducted in tropical low-income countries (LICs), where the seasons are generally defined by rainfall [2–3]. Data on seasonal variations are quite scarce, but deeper insight into the impact of seasonality on each pathogen’s epidemiology may offer tools for prevention and even guide treatment.

In Guinea-Bissau, like in all equatorial tropical countries, the temperature differences over the year are minimal, mostly with averages ranging between 28 and 32 degrees. There is, however, a monsoonal-like rainy season from June to October and a dry season from November to May, with monthly rainfall averages of 70–600mm and 0–20mm, respectively [4]. The data remain limited, but weather conditions may have a considerable effect on the types of prevailing pathogens. For example, in the Global Enteric Multicenter Study (GEMS) conducted in four sub-Saharan African countries (Kenya, Mali, Mozambique, and Gambia) and three South Asian (Bangladesh, India, and Pakistan) [5], Chao et al. report rotavirus prevailing in the drier and bacterial pathogens in the wetter months [6]. Only a few studies have explored seasonality of diarrhoeagenic pathogens among children in Guinea-Bissau [7–9], mainly focusing on rotavirus and *Cryptosporidium*. In the absence of national surveillance programmes, academic research provides despite its limitations, for example, with respect to continuity over time, an important source of data on the seasonality of the agents causing diarrhoea in LICs.

To investigate the associations between various diarrhoeal pathogens and seasonality in childhood diarrhoea, we explored a large variety of bacterial, viral and parasitic agents among children with and without diarrhoea during both rainy and dry seasons. Moreover, while we previously observed differences in the occurrence of various pathogens between infants and young children [10], we now scrutinized whether these differences were seasonally impacted. Characterizing such epidemiology of diarrhoeal pathogens can enhance clinical approaches for diagnostics and treatment by season. In addition, defining the weather conditions for each pathogen should enable identification of pathways of pathogen spread and prediction of large outbreaks.

## Materials and methods

### Ethics statement

The study protocol was approved by the Comité Nacional de Ética na Saúde, Instituto Nacional de Saúde Pública, Guinea-Bissau (No: 031/CNES/2010). As described in our previous report in the same study setting [10], the children’s parents or caregivers were informed about the aim of the study and they signed a written informed consent form.

### Study population and sample material

**Fig 1:**
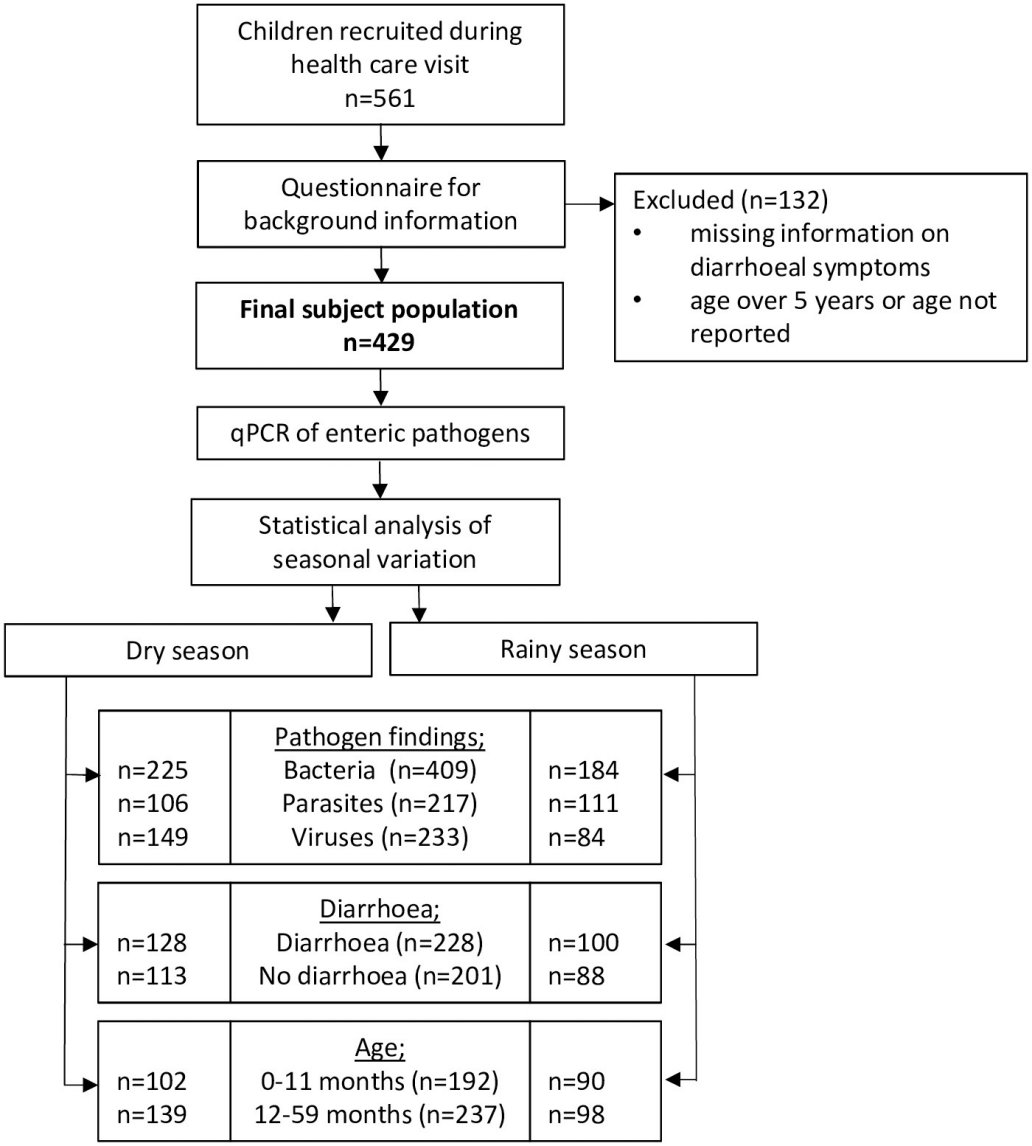
Flow chart of the study. The final study population comprised 429 children; 424 of these were tested for bacterial, 397 for viral, and 426 for parasitic pathogens.

This study was a secondary analysis of data from an unmatched, health facility-based case-control study, the procedures and primary findings of which have been reported elsewhere [10]. The study was conducted at the Bandim Health and Demographic Surveillance Site (HDSS) serving an area of 16 km^2^ in suburban Bissau, the capital of Guinea-Bissau (www.bandim.org). A total of 561 children were recruited between November 2010 and October 2012 from among consecutive patients (excluding weekends and night-time) seeking medical care at the Bandim Health Centre, which is one of the three health centres in the Bandim HDSS (Fig 1). The study population was selected to cover children with and without diarrhoea in two age groups: infants (0–11 months) and young children (12–59 months). The inclusion criteria comprised age less than five years and information available on the study nurse’s interview form concerning presence/lack of diarrhoea at the time of sampling; those with ongoing diarrhoea were included in the diarrhoea group and those with no diarrhoeal symptoms over the past seven days in the control group. Children requiring hospital care were not eligible.

### Definition of children’s diarrhoea

Diarrhoea was defined by WHO criteria [11] as passage of three or more loose or liquid stools per day or, more frequently than is normal for the individual. The children were defined as not having diarrhoea if they had not had diarrhoea during the last seven days prior to attending the health centre.

### Weather data, definition of dry and rainy season and age stratification

Monthly averages of daily temperature and precipitation for Bissau were extracted from WorldWeatherOnline.com [4] and visualized by plotting them in time series graphs alongside taxon- and species-specific enteropathogen detection rates. The rainy season was defined as the months in which the average rainfall exceeded 50 mm (June to October) and the dry season as months it did not exceed this limit (November to May). The data were also analysed by age group, allowing comparisons between infants (0–11 months) and small children (12–59 months).

### PCR for the detection of diarrhoeal pathogens

PCRs for the detection of diarrhoeal pathogens were described in our previous study [10]. Briefly, we used qPCR assays with a large coverage of pathogens: nine enteric bacteria, *Campylobacter*, enteroaggregative (EAEC), enterohaemorrhagic (EHEC), enteroinvasive (EIEC)/*Shigella*, enteropathogenic (EPEC), enterotoxigenic *E*. *coli* (ETEC), *Salmonella*, *Yersinia* and *Vibrio cholerae* O1 [12]; five viruses: adenovirus 40 and 41, astrovirus, norovirus GI and GII, rotavirus A and sapovirus (Amplidiag Viral GE; Mobidiag Ltd, Helsinki, Finland); and four parasites: *Cryptosporidium*, *Dientamoeba fragilis*, *Entamoeba histolytica* and *Giardia duodenalis* (Amplidiag Stool Parasites; Mobidiag Ltd, Helsinki, Finland).

### Statistical analysis

Pearson’s chi-square test or Fisher’s exact test were used to compare categorical variables when applicable. Statistical significance was defined as p<0.05 or ORs with 95% CIs ranging either above or below 1. Exact binomial 95% CIs for proportions were calculated. The increase/decrease of pathogen findings by 100 mm of rainfall was analysed by logistic regression. The unadjusted population attributable fraction (AF) was defined as $AF=\frac{Pr\;\left(disease\right)\;-Pr(disease|not\;exposed)}{Pr\;\left(disease\right)\;}$ which by the Bayesian formula equals to $Pr\;(exposed\;|\;disease)\left(1-\frac{1}{RR}\right)$, in which RR is the relative risk of the disease. We used logistic regression with robust standard error estimators to evaluate attributable fractions by predicting the number of cases needed for calculating the estimate of AF and its uncertainty (standard error). The unadjusted AF is estimated by $\frac{observed\;no.\;ill-expected\;no.\;ill\;on\;removal\;of\;the\;exposure\;}{observed\;no.\;ill}$ [13]. The statistical analyses were carried out using SPSS 22 software (IBM Corp., Armonk, NY) and Stata 17.0 (StataCorp, College Station, TX).

## Results

### Study population

Of the 561 children recruited, 429 met the inclusion criteria, 228 of them having diarrhoea and 201 not having the disease. Of all children, 211 (50.8%) were females, 193 (45.0%) infants (0–11 months), and 236 (55.0%) young children (12–59 months). Flow chart of study conduct is shown in Fig 1.

### Seasonality of diarrhoeal pathogens

**Fig 2:**
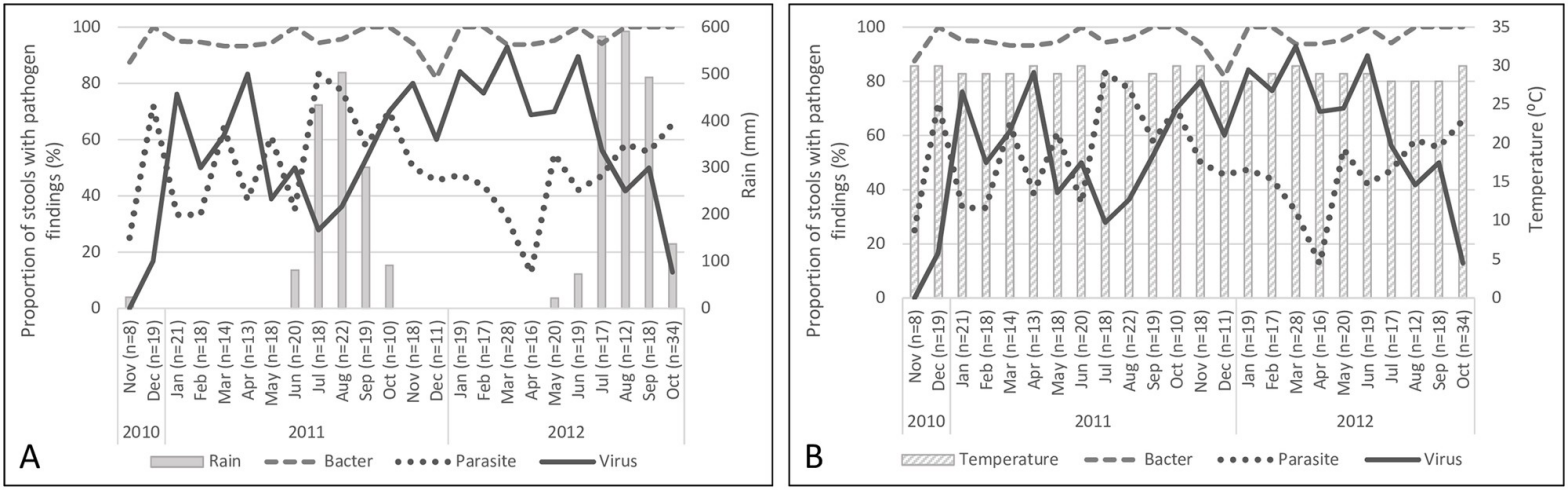
Seasonal variation of diarrhoeal pathogens identified over the 24-month study period with respect to rainfall [4] (A) and temperature (B). Monthly proportions given for those with samples positive for bacterial, parasitic, or viral diarrhoeal pathogens.

**Fig 3:**
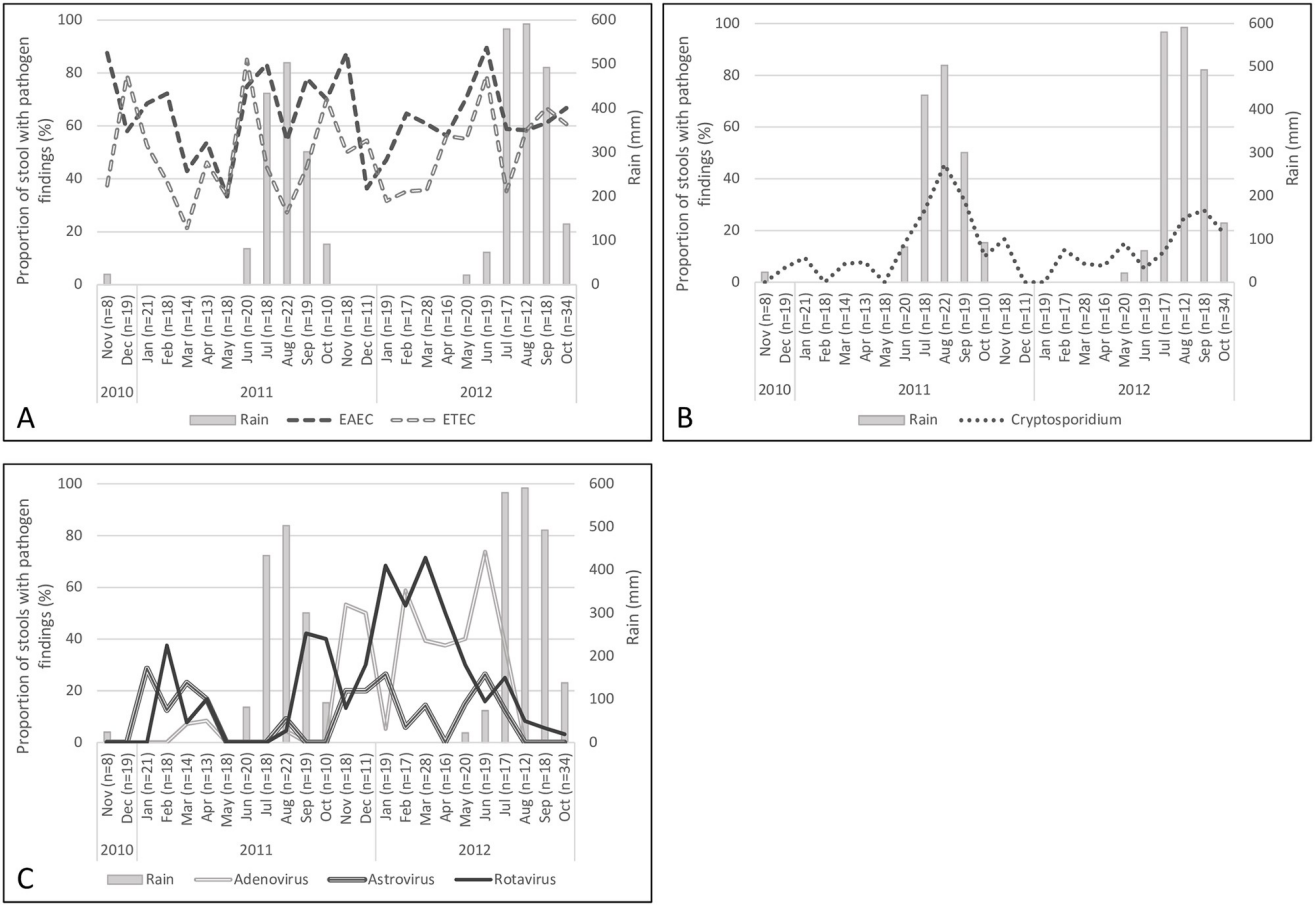
Seasonal variation of diarrhoeal pathogens with respect to rainfall [4]. For the six pathogens showing seasonal variation in incidence, data are shown as monthly proportions of samples positive for A) bacterial (EAEC, ETEC), B) parasitic (*Cryptosporidium)*, and C) viral pathogens (adeno-, astro-, and rotavirus). Details of all pathogen findings are shown in S1 Fig. Numbers of children are indicated in parentheses for each month.

As indicated in our previous report, coinfections with multiple pathogen species types (bacteria, viruses, parasites) were common [10]. Overall, findings of viral pathogens in the total study population were most common in the dry season (45.4% in the rainy versus 70.3% in dry season, p<0.001) and parasitic pathogens in the rainy season (59.4% versus 44.4%, p = 0.002). With bacterial pathogens, the rates were very high regardless of season (98.4% versus 94.9%) (Table 1 and Fig 2). These differences were mostly explained by six individual pathogens showing seasonal differences in prevalence (Table 1): EAEC, ETEC, *Cryptosporidium*, adenovirus, astrovirus and rotavirus (Fig 3); a seasonal variation was also observed with *Campylobacter*, yet it did not reach statistical significance (58.3% versus 48.9%, p = 0.056). EAEC (69.5% versus 59.9%, p = 0.041), ETEC (56.7% versus 45.1%, p = 0.018) and *Cryptosporidium* (22.5% versus 7.1%, p<0.001) were found more frequently during the rainy season, and rotavirus (12.4% versus 33.0%, p<0.001), astrovirus (4.9% versus 14.6%, p = 0.001), and adenovirus (11.4% versus 24.5%, p = 0.001) during the dry season. For the other pathogens, no clear differences were seen between seasons. A separate analysis by the amount of rainfall showed that 100 mm per month significantly increased the number of *Campylobacter* (p = 0.026) and *Cryptosporidium* (p<0.001) findings, and correspondingly decreased the number of virus findings (p<0.001), mainly adenovirus, astrovirus and rotavirus (Table 1).

**Table 1:** Microbial findings among 429 Guinea-Bissauan children under five years of age during dry (November to May) and rainy (June to October) seasons*.

	Total n (%)	Dry season n (%)	95% CI (%)	Rainy season n (%)	95% CI (%)	Rainy versus dry season OR (95% CI) p-value		Impact of 100 mm monthly rainfall on pathogen findings OR (95% CI) p-value	
**All subjects**	429 (100)	241 (56.2)		188 (43.8)					
**Children with diarrhoea**	228 (53.1)	128 (53.1)		100 (53.2)		1.0 (0.7–1.5)	0.987	1.0 (0.9–1.1)	0.856
**Any pathogen**	422 (98.4)	236 (97.9)		186 (98.9)		2.0 (0.4–10.3)	0.474		
**Any bacteria** ^a^	409 (96.5)	225 (94.9)	91.3–97.3	184 (98.4)	95.4–99.7	3.3 (0.9–11.8)	0.056	1.1 (0.8–1.5)	0.536
*Campylobacter*	225 (53.1)	116 (48.9)	42.8–55.9	109 (58.3)	50.3–64.8	1.5 (1.0–2.1)	0.056	1.1 (1.0–1.2)	**0.026**
EAEC	272 (64.2)	142 (59.9)	53.4–66.3	130 (69.5)	62.8–76.3	1.5 (1.0–2.3)	**0.041**	1.0 (0.9–1.1)	0.704
EHEC	6 (1.4)	3 (1.3)	NA	3 (1.6)	NA	1.3 (0.3–6.4)	1.000	NA	NA
EIEC/*Shigella*	98 (23.1)	48 (20.3)	15.1–25.7	50 (26.7)	20.8–33.9	1.4 (0.9–2.3)	0.116	1.0 (0.9–1.2)	0.435
EPEC	266 (62.7)	143 (60.3)	53.4–66.3	123 (65.8)	58.4–72.4	1.3 (0.8–1.9)	0.250	1.0 (0.9–1.1)	0.441
ETEC	213 (50.2)	107 (45.1)	39.0–52.1	106 (56.7)	49.8–64.3	1.6 (1.1–2.3)	**0.018**	1.0 (0.9–1.1)	0.565
*Salmonella*	11 (2.6)	5 (2.1)	NA	6 (3.2)	NA	1.5 (0.5–5.1)	0.342	NA	NA
*V*. *cholerae*	2 (0.5)	1 (0.4)	NA	1 (0.5)	NA	1.3 (0.1–20.4)	1.000	NA	NA
*Yersinia*	3 (0.7)	2 (0.8)	NA	1 (0.5)	NA	0.6 (0.1–7.0)	1.000	NA	NA
**Any parasites** ^b^	217 (50.9)	106 (44.4)	37.5–50.5	111 (59.4)	52.4–66.8	1.8 (1.2–2.7)	**0.002**	1.2 (1.1–1.3)	**0.001**
*Cryptosporidium*	59 (13.8)	17 (7.1)	4.2–11.2	42 (22.5)	17.9–30.5	3.8 (2.1–6.9)	**<0.001**	1.4 (1.2–1.5)	**<0.001**
*D*. *fragilis*	43 (10.7)	26 (11.9)	8.0–17.1	17 (9.2)	5.4–14.2	0.8 (0.4–1.4)	0.385	1.1 (0.9–1.2)	0.438
*E*. *histolytica*	3 (0.7)	0 (0.0)	NA	3 (1.6)	NA	NA	0.084	NA	NA
*Giardia*	159 (37.3)	88 (36.8)	30.2–42.8	71 (38.0)	30.1–44.4	1.1 (0.7–1.6)	0.808	1.0 (0.9–1.1)	0.474
**Any virus** ^c^	233 (58.7)	149 (70.3)	64.3–77.0	84 (45.4)	37.1–51.8	0.4 (0.2–0.5)	**<0.001**	0.8 (0.7–0.9)	**<0.001**
Adenovirus 40, 42	73 (18.4)	52 (24.5)	19.1–31.2	21 (11.4)	7.1–16.7	0.4 (0.2–0.7)	**0.001**	0.8 (0.6–0.9)	**0.005**
Astrovirus	40 (10.1)	31 (14.6)	10.3–20.3	9 (4.9)	2.2–8.9	0.3 (0.1–0.6)	**0.001**	0.7 (0.6–1.0)	**0.034**
Norovirus GI/GII	92 (23.2)	48 (22.6)	17.4–29.1	44 (23.8)	17.2–29.7	1.1 (0.7–1.7)	0.788	1.0 (0.9–1.1)	0.871
Rotavirus A	93 (23.4)	70 (33.0)	27.0–40.2	23 (12.4)	8.0–17.9	0.3 (0.2–0.5)	**<0.001**	0.8 (0.6–0.9)	**0.001**
Sapovirus	29 (7.3)	20 (9.4)	5.9–14.3	9 (4.9)	2.2–8.9	0.5 (0.2–1.1)	0.081	0.9 (0.7–1.1)	0.426

Data is missing, n (%):

^a)^ 5 (1.2),

^b)^ 3 (0.7),

^c)^ 32 (7.5)

CI = confidence interval; NA = not applicable; OR = odds ratio; **bolding** indicates statistically significant at p<0.05 (Pearson χ^2^ test or Fisher´s exact test)

*Coinfections with multiple pathogen species were observed in 97.4% of samples collected in the rainy and 92.1% in the dry season (p = 0.017; OR 3.2 95% CI 1.2–8.7). Viruses and bacteria were found in 19.5% versus 41.2% (p<0.001; OR 0.3 95% CI 0.2–0.5), bacteria and parasites in 34.1% versus 13.7% (0<0.001; OR 3.2 95% CI 2.0–5.4), viruses and parasites in 0% versus 1.0% (p = 0.500; OR NA), and all three in 24.9% versus 27.5% (p = 0.562; OR 0.9 95% CI 0.6–1.4), respectively.

The temperature In Guinea-Bissau does not vary much during the year and, thus, the variation in the rates of the various pathogen findings appears not to depend on changes in temperature (Fig 2b).

### Seasonality of diarrhoeal pathogens by age group

In both age groups, an association was found between the findings of *Cryptosporidium* and rainy season (Table 2). As for the viral pathogens, an association with the dry season was observed for rotavirus among infants (dry versus rainy season: 40.0% versus 11.2%, p<0.0019, and young children (27.9% versus 13.5%, p = 0.011), for astrovirus among infants (16.7% versus 5.6%, p = 0.019) and young children (13.1% versus 4.2%, p = 0.023) and for adenovirus among infants (31.1% versus 10.1%, p = 0.001). For bacterial pathogens, we observed an association with the rainy season in the following age groups: for *Campylobacter* among infants (rainy versus dry season: 58.4% versus 43.6%, p = 0.041), for ETEC among infants (62.9% versus 47.5%, p = 0.033) and for EAEC among young children (66.3% versus 50.0%, p = 0.013).

**Table 2:** Effect of seasonality on microbial findings by age group.

	Total n (%)	0–11 months				12–59 months			
		Rainy season n (%)	Dry season n (%)	Rainy versus dry season OR (95% CI) p-value		Rainy season n (%)	Dry season n (%)	Rainy versus dry season OR (95% CI) p-value	
**All**	429 (100)	90 (46.9)	102 (52.1)			98 (41.4)	139 (58.6)		
**Children with diarrhoea**	228 (53.1)	42 (46.7)	57 (55.9)	0.7 (0.4–1.2)	0.202	58 (59.2)	71 (51.1)	1.4 (0.8–2.3)	0.217
**Any pathogen**	422 (98.4)	88 (97.8)	101 (99.0)	0.4 (0.0–4.9)	0.601	98 (100)	135 (97.1)	NA	0.144
**Any bacteria** ^a^	409 (96.5)	87 (97.8)	97 (96.0)	1.8 (0.3–10.0)	0.686	97 (99.0)	128 (94.1)	6.1 (0.7–49.3)	0.083
*Campylobacter*	225 (53.1)	52 (58.4)	44 (43.6)	1.8 (1.0–3.2)	**0.041**	57 (58.2)	72 (52.9)	1.2 (0.7–2.1)	0.428
EAEC	272 (64.2)	65 (73.0)	74 (73.3)	1.0 (0.5–1.9)	0.971	65 (66.3)	68 (50.0)	2.0 (1.2–3.4)	**0.013**
EHEC	6 (1.4)	1 (1.1)	0 (0.0)	NA	0.468	2 (2.0)	3 (2.2)	0.9 (0.2–5.6)	1.000
EIEC/*Shigella*	98 (23.1)	14 (15.7)	14 (13.9)	1.2 (0.5–2.6)	0.717	36 (36.7)	34 (25.0)	1.7 (1.0–3.1)	0.053
EPEC	266 (62.7)	61 (68.5)	62 (61.4)	1.4 (0.8–2.5)	0.303	62 (63.3)	81 (59.6)	1.2 (0.7–2.0)	0.566
ETEC	213 (50.2)	56 (62.9)	48 (47.5)	1.9 (1.0–3.4)	**0.033**	50 (51.0)	59 (43.4)	1.4 (0.8–2.3)	0.248
*Salmonella*	11 (2.6)	4 (4.5)	4 (4.0)	1.1 (0.3–4.7)	1.000	2 (2.0)	1 (0.7)	2.8 (0.3–4.7)	0.573
*V*. *cholerae*	2 (0.5)	0 (0.0)	1 (1.0)	NA	1.000	1 (1.0)	0 (0.0)	NA	0.419
*Yersinia*	3 (0.7)	1 (1.1)	1 (1.0)	1.1 (0.1–18.4)	1.000	0 (0.0)	1 (0.7)	NA	1.000
**Any parasites** ^b^	217 (50.9)	46 (51.1)	29 (28.7)	2.6 (1.4–4.7)	**0.002**	65 (67.0)	77 (55.8)	1.6 (0.9–2.8)	0.084
*Cryptosporidium*	59 (13.8)	23 (25.6)	8 (7.9)	4.0 (1.7–9.5)	**0.001**	19 (19.6)	9 (6.5)	3.5 (1.5–8.1)	**0.002**
*D*. *fragilis*	43 (10.7)	2 (2.3)	7 (7.5)	0.3 (0.1–1.4)	0.170	15 (15.6)	19 (15.2)	1.0 (0.5–2.2)	0.931
*E*. *histolytica*	3 (0.7)	1 (1.1)	0 (0.0)	NA	0.471	2 (2.1)	0 (0.0)	NA	0.169
*Giardia*	159 (37.3)	24 (26.7)	21 (20.8)	1.4 (0.7–2.7)	0.340	47 (48.5)	67 (48.6)	1.0 (0.6–1.7)	0.988
**Any viruses** ^c^	233 (58.7)	44 (49.4)	72 (80.0)	0.2 (0.1–0.5)	**<0.001**	40 (41.7)	77 (63.1)	0.4 (0.2–0.7)	**0.002**
Adenovirus 40, 42	73 (18.4)	9 (10.1)	28 (31.1)	0.3 (0.1–0.6)	**0.001**	12 (12.5)	24 (19.7)	0.6 (0.3–1.2)	0.157
Astrovirus	40 (10.1)	5 (5.6)	15 (16.7)	0.3 (0.1–0.9)	**0.019**	4 (4.2)	16 (13.1)	0.3 (0.1–0.9)	**0.023**
Norovirus GI/GII	92 (23.2)	26 (29.2)	26 (28.9)	1.0 (0.5–1.9)	0.962	18 (18.8)	22 (18.0)	1.0 (0.5–2.1)	0.892
Rotavirus A	93 (23.4)	10 (11.2)	36 (40.0)	0.2 (0.1–0.4)	**<0.001**	13 (13.5)	34 (27.9)	0.4 (0.2–0.8)	**0.011**
Sapovirus	29 (7.3)	4 (4.5)	10 (11.1)	0.4 (0.1–1.2)	0.099	5 (5.2)	10 (8.2)	0.6 (0.2–1.9)	0.387

Data is missing, n (%):

^a)^ 5 (1.2),

^b)^ 3 (0.7),

^c)^ 32 (7.5)

CI = confidence interval; NA = not applicable; OR = odds ratio; **bolding** indicates statistically significant at p<0.05 (Pearson χ^2^ test or Fisher´s exact test)

### Impact of season on pathogen findings among those with and without diarrhoea

We compared those with symptomatic diarrhoea to those without diarrhoea separately during each of the two seasons. Statistically significant differences were seen for viruses; they were more prevalent among those with diarrhoea than those without the disease during both seasons (Table 3). A tendency was seen for practically all viruses, but statistical significance was found for norovirus (rainy season: diarrhoea 29.6% versus no diarrhoea 17.2%; dry season: diarrhoea 28.9% with no diarrhoea 15.5%) and astrovirus (rainy season: diarrhoea 8.2% versus no diarrhoea 1.1%; dry season: diarrhoea 19.3% versus no diarrhoea 9.2%) in both seasons. Of the parasites, the higher rates of cases among those with diarrhoea were seen with *Cryptosporidium* during both seasons. As for bacteria, the only significant findings were those for ETEC, which showed in the dry season higher rates among those with diarrhoea than those without it (rainy season: diarrhoea 57.0% versus no diarrhoea 56.3%; dry season: diarrhoea 52.8% versus no diarrhoea 36.4%). In the rainy season, most of the diarrhoea cases were attributable to norovirus (8%) and *Cryptosporidium* (6%) and in the dry season to ETEC (13%) and norovirus (8%) (Table 3).

**Table 3:** Diarrhoeal pathogens in children with and without diarrhoea during the dry and rainy seasons.

	Total n (%)	Dry season					Rainy season				
		No diarrhoea n (%)	Diarrhoea n (%)	Diarrhoea versus no diarrhoea OR (95% CI) p-value		AF (95% CI)	No diarrhoea n (%)	Diarrhoea n (%)	Diarrhoea versus no diarrhoea OR (95% CI) p-value		AF (95% CI)
**Total**	429 (100)	113 (46.9)	128 (53.1)				88 (46.8)	100 (53.2)			
**Any pathogen**	422 (98.4)	109 (96.5)	127 (99.2)	4.7 (0.5–42.3)	0.189		87 (98.9)	99 (99.0)	1.1 (0.1–18.5)	1.000	
**Any bacteria** ^a^	409 (96.5)	103 (93.6)	122 (96.1)	1.7 (0.5–5.4)	0.395	-0.2 (-0.5–0.6)	85 (97.7)	99 (99.0)	2.3 (0.2–26.1)	0.598	0.4 (-2.1–0.9)
*Campylobacter*	225 (53.1)	56 (50.9)	60 (47.2)	0.9 (0.5–1.4)	0.574	-0.0 (-0.2–0.1)	51 (58.6)	58 (58.0)	1.0 (0.5–1.4)	0.932	0.1 (-0.2–0.2)
EAEC	272 (64.2)	71 (64.5)	71 (55.9)	0.7 (0.4–1.2)	0.176	-0.1 (-0.3–0.0)	63 (72.4)	67 (67.0)	0.8 (0.4–1.5)	0.422	-0.1 (-0.3–0.1)
EHEC	6 (1.4)	3 (2.7)	0 (0.0)	NA	0.099	NA	2 (2.3)	1 (1.0)	0.4 (0.0–4.8)	0.598	NA
EIEC/*Shigella*	98 (23.1)	17 (15.5)	31 (24.4)	1.8 (0.9–3.4)	0.087	0.1 (-0.0–0.1)	19 (21.8)	31 (31.0)	1.6 (0.8–3.1)	0.158	0.1 (-0.0–0.1)
EPEC	266 (62.7)	66 (60.0)	77 (60.6)	1.0 (0.6–1.7)	0.921	0.0 (-0.1–0.2)	60 (69.0)	63 (63.0)	0.8 (0.4–1.4)	0.391	-0.1 (-0.3–0.1)
ETEC	213 (50.2)	40 (36.4)	67 (52.8)	2.0 (1.2–3.3)	**0.011**	0.1 (0.0–0.2)	49 (56.3)	57 (57.0)	1.0 (0.6–1.8)	0.926	-0.0 (-0.2–0.1)
*Salmonella*	11 (2.6)	3 (2.7)	2 (1.6)	0.6 (0.1–3.5)	0.665	NA	3 (3.4)	3 (3.0)	0.9 (0.2–4.4)	1.000	NA
*V*. *cholerae*	2 (0.5)	0 (0.0)	1 (0.8)	NA	1.000	NA	0 (0.0)	1 (1.0)	NA	1.000	NA
*Yersinia*	3 (0.7)	1 (0.9)	1 (0.8)	0.9 (0.1–14.0)	1.000	NA	0 (0.0)	1 (1.0)	NA	1.000	NA
**Any parasites** ^b^	217 (50.9)	49 (43.8)	57 (44.9)	1.0 (0.6–1.7)	0.860	0.0 (-0.1–0.1)	48 (55.2)	63 (63.0)	1.4 (0.8–2.5)	0.277	0.1 (-0.1–0.2)
*Cryptosporidium*	59 (13.8)	3 (2.7)	14 (11.0)	4.5 (1.3–16.0)	**0.012**	0.0 (0.0–0.1)	14 (16.1)	28 (28.0)	2.0 (1.0–4.2)	0.052	0.1 (-0.0–0.1)
*D*. *fragilis*	43 (10.7)	13 (12.9)	13 (11.1)	0.8 (0.4–1.9)	0.689	-0.0 (-0.1–0.1)	7 (8.0)	10 (10.3)	1.3 (0.5–3.6)	0.597	0.0 (-0.0–0.1)
*E*. *histolytica*	3 (0.7)	0 (0.0)	0 (0.0)	NA	NA	NA	3 (3.4)	0 (0.0)	NA	0.099	NA
*Giardia*	159 (37.3)	43 (38.4)	45 (35.4)	0.9 (0.5–1.5)	0.636	-0.0 (-0.1–0.1)	31 (35.6)	40 (40.0)	1.2 (0.7–2.2)	0.539	0.0 (-0.1–0.1)
**Any viruses** ^c^	233 (58.7)	59 (60.2)	90 (78.9)	2.5 (1.4–4.5)	**0.003**	0.3 (0.1–0.4)	31 (35.6)	53 (54.1)	2.1 (1.2–3.8)	**0.012**	0.2 (0.0–0.3)
Adenovirus 40, 42	73 (18.4)	27 (27.6)	25 (21.9)	0.7 (0.4–1.4)	0.343	-0.0 (-0.1–0.0)	6 (6.9)	15 (15.3)	2.4 (0.9–6.6)	0.072	0.0 (-0.0–0.1)
Astrovirus	40 (10.1)	9 (9.2)	22 (19.3)	2.4 (1.0–5.4)	**0.038**	0.1 (0.0–0.1)	1 (1.1)	8 (8.2)	7.6 (0.9–62.4)	**0.037**	0.0 (0.0–0.1)
Norovirus GI/GII	92 (23.2)	15 (15.3)	33 (28.9)	2.3 (1.1–4.5)	**0.018**	0.1 (0.0–0.1)	15 (17.2)	29 (29.6)	2.0 (1.0–4.1)	**0.049**	0.1 (0.0–0.2)
Rotavirus A	93 (23.4)	30 (30.6)	40 (35.1)	1.2 (0.7–2.2)	0.490	0.0 (-0.1–0.1)	10 (11.5)	13 (13.3)	1.2 (0.5–2.8)	0.716	0.0 (-0.0–0.1)
Sapovirus	29 (7.3)	8 (8.2)	12 (10.5)	1.3 (0.5–3.4)	0.557	0.0 (-0.0–0.1)	2 (2.3)	7 (7.1)	3.3 (0.7–16.2)	0.176	0.0 (-0.0–0.0)

Data missing, n (%):

^a)^ 5 (1.2),

^b)^ 3 (0.7),

^c)^ 32 (7.5)

AF = attributable fraction; CI = confidence interval; NA = not applicable; OR = odds ratio; **bolding** indicates statistically significant at p<0.05 (Pearson χ^2^ test or Fisher´s exact test)

## Discussion

The prevalence of individual pathogens differed by season and age group, and according to whether or not the children had diarrhoea. *Cryptosporidium* was most prevalent during the rainy season, whereas viral pathogens (adenovirus, astrovirus and rotavirus) were most frequently found during the dry season. Bacterial pathogens showed a tendency towards slightly higher rates in the rainy (98.4%) than the dry (94.9%) season, but overall, EAEC, EPEC, ETEC and *Campylobacter* were the most prevalent pathogens in both seasons.

### Impact of temperature on occurrence of diarrhoea and diarrhoeal pathogens

Many research findings from high-income countries connect diarrhoeal pathogens with temperature rather than rainfall [1,14–17]. In their systematic literature review covering 59 studies published 1974–2010, Philipsborn et al. report an association between high temperature and findings of diarrhoeagenic *E*. *coli* (DEC). They depict a growth of 8% in DEC incidence for each 1°C increase in monthly temperature; however, nontropical industrialized countries were also included in the review [14]. The impact of temperature may be related to various factors, such as enhanced pathogen survival, increased pathogen load in (animal) reservoirs, and prolonged transmission seasons [14] or differences in expression of virulence genes [1]. Since the temperature in Guinea-Bissau does not vary much by season [4], no major temperature-related impact appeared likely.

### Impact of rainfall on occurrence of diarrhoea

As there are substantial seasonal variations in rainfall in Guinea-Bissau, we also expected to see such variation in diarrhoea cases. Indeed, Colombatti et al. report severe faecal contamination of drinking water from taps and wells during the rainy season in the country [18]. In the literature, however, the impact of rainfall on the occurrence of diarrhoea remains somewhat controversial [19–20]. In many African countries, such as Botswana [19] Mozambique [20] and Niger [21], rainfall has been associated with diarrhoea. A Ghanaian investigation found diarrhoea risk to grow 5.1% for every 10mm increase in rainfall [21]. Flooding and heavy precipitation may flush faecal and other waste into areas where humans easily become exposed [22]. The seasons may also affect water quality and access to water as well as various wildlife and agricultural activities [20,23–24]. Some studies suggest that in households with poor-quality water sources, the risk of diarrhoea grows with heavy rainfall, while dry conditions decrease it [20–22,25]. On the other hand, during the dry season, drought and smaller rainfall can increase the proportion of wastewater in surface water, leading to greater pathogen concentration in both drinking and irrigation water [26]. In dry conditions water sources are also more likely to be shared by human and animal populations [1]. Some studies [19,27–28] relate the higher rate of pathogen findings in the dry season to decreased access to handwashing [22]. Indeed, handwashing with soap can bring about a reduction of up to 27% of diarrhoeal cases [29].

Importantly, only about 15% of Guinea-Bissauan inhabitants have been estimated to have access to adequate sanitation, and only 2% to basic waste management services (waste separation, treatment and safe disposal) [30]. Although we did not see a change in the rates of diarrhoea per se, the prevalence of various pathogen findings changed by season, as discussed below.

### Impact of season on occurrence of diarrhoeal pathogens

#### *Cryptosporidium* common during rainy season

According with previous studies [2,14,21], the pathogen most strongly associated with the rainy season was *Cryptosporidium*, a major cause of waterborne gastrointestinal disease with potential to large outbreaks [31]. An earlier study carried out in Guinea-Bissau 1991–1997 also connects the peak in the prevalence of *Cryptosporidium* with wetter times of the year; very few cases having this pathogen have been reported for dry seasons [7]. Indeed, cryptosporidiosis incidence has been suggested to be related to contamination of water supplies through heavy rainfall [1–2,6,32]. This tallies with our number of detected cases increasing sharply in July, peaking in August when the rains are heaviest, and decreasing again with the sparse precipitation in October (Fig 3).

As for the other parasites in our study, we saw no signs of seasonality for *Giardia* and *Dientamoeba*. This difference between the various parasites seems logical, since *Giardia* and *Dientamoeba* are often transmitted directly via the faecal-oral route rather than water like *Cryptosporidium*. Their spread can be largely controlled by improving hygiene and sanitation conditions, according with their non-seasonal occurrence [33–34].

#### Bacterial pathogens prevalent all year

Bacterial pathogens proved very common throughout the year (rainy season 98.4% versus dry season 94.9%; p = 0.056) (Table 1). Findings of *Campylobacter* increased by the amount of rainfall. Moreover, ETEC and EAEC were associated with the rainy season for all children (Table 1). The extensive cohort study of ETEC infections conducted by Steinsland et al. in Guinea-Bissau 1996 and 1998 monitored children from birth to two years of age with weekly faecal sampling. Observing an increase in ETEC during the 1997 rainy season, they concluded that rainy season epidemics may be annual [35]. This accords with our ETEC data for infants: they had more ETEC findings during the rainy season, whereas no seasonal difference was seen in the older age group, a possible consequence of gradually developing intestinal immunity to this highly common pathogen. A similar seasonal trend was also seen for *Campylobacter* infections. In industrialized countries, *Campylobacter* infections peak over warmer summer months, but many African studies show no strong seasonal trends [36–37]. However, an earlier investigation carried out in Guinea-Bissau 1987–88 [38] accords with our findings. The seasonal difference for EAEC was only seen among young children, but not infants, though (Table 2). The reason for this remains unknown.

#### Viral pathogens common during dry season

According with earlier research [2,14,21,39–40], decrease in the amount of rainfall and the dry season were strongly associated with findings of adenovirus, astrovirus, and rotavirus (Fig 2). In fact, their incidences dropped dramatically already at the beginning of the rainy season (Fig 3). These findings agree with a study exploring rotavirus diarrhoea in Guinea-Bissau between 1996 and 1998 [9]. The virus’s high prevalence in the dry season has been suggested to be attributable to aridity of soil increasing aerial transport of dried faecal material in the form of droplet nuclei. In addition, dust may serve as a vehicle for virus particles [26,41].

Unlike the case of the other viruses in our study, norovirus findings were evenly distributed throughout the year. This difference may be ascribed to noroviruses being transmitted not only by the faecal-oral route but also aerosols from vomiting. Noroviruses’ seasonality has barely been studied in LICs, but among Malawian children aged 18 months or younger, noroviruses have been reported to prevail in the rainy season [42]. In cooler regions, norovirus infections have been found mainly to be related to colder temperatures (“winter vomiting disease”) [43]. Indeed, the literature suggests low temperature (-5–20°C) and low relative humidity (10–60%) to be associated with the occurrence of norovirus epidemics worldwide [44].

#### Impact of participants’ ages on seasonal differences

The associations between age, season and diarrhoea are complicated. For example, highly prevalent pathogens encountered early in life may no longer cause diarrhoea for older children due to pre-existing intestinal immunity elicited by previous exposures [5,36]. The frequency and load of pathogens to which children become exposed may vary by age and season, resulting in differences between pathogen findings for infants and young children. Indeed, when scrutinizing the seasonality of the pathogens in the two age groups, the association appeared stronger among infants than young children. Among infants, seasonal differences were observed for *Campylobacter*, ETEC, *Cryptosporidium*, adeno-, astro- and rotavirus, while among young children such differences were seen for EAEC, *Cryptosporidium*, astro-, and rotavirus. Our data encourage further research, particularly since we found no recent studies that would compare pathogens’ seasonality and children’s ages.

#### Diarrhoeal pathogens found in dry and rainy seasons among children with and without diarrhoea

Some of the previous studies from West Africa report DEC rates (1–15%) lower than ours (23–64%) [45–47]. Apart from differences in study sites and PCR cut-off values, the explanation may be merely methodological: we detected DEC directly from stools by PCR, while they used culture-based methods an approach known to be substantially less sensitive [48]. Our rates concur with those reported among local children in LICs [49–50] and traveller studies conducted in West Africa [51–52], all assessing the pathogens directly from stools by PCR.

Our prevalence analysis of various pathogens and diarrhoeal symptoms during the dry or rainy season scrutinizes a subject barely covered in scientific literature. Our data does not show many significant differences apart from greater prevalence of astrovirus, norovirus and *Cryptosporidium* among those with diarrhoea than those without in both seasons. It is noteworthy that these pathogens all have low infectious doses. As for bacterial pathogens, the only statistically significant finding was that of ETEC during the dry season; it proved more prevalent among those with diarrhoeal symptoms than those without any—despite ETEC’s greater occurrence in the rainy season. This phenomenon may be ascribed to ETEC’s high infectious doses: as the inoculum may be diluted during the rainy season, many of the exposed individuals do not catch infectious doses. The finding may also be explained by the presence of ETEC in biofilms in water tanks used during the dry season for irrigation or washing fresh vegetables and fruits [53]. Biofilms may serve as reservoirs for this group of *E*. *coli* [54]. Bacterial recovery from dry-surface biofilm has been shown to amount to 100% [55]. The relatively low attributable fractions for practically all single pathogens reveal the polymicrobial character of childhood diarrhoea in West Africa, highlighting the preventive value of hygiene measures.

### Limitations

The main limitation in our study was the restricted number of cases, which prevented some of the potential analyses used in other studies [21,26,40]: with higher numbers of patients and longer follow-up period, more flexible approaches have been used for characterizing the timing, amplitude and number of annual seasonal peaks in enteropathogen detection [40,56]. We now opted, instead, to use attributable fractions to express the proportions of the observed pathogens attributable to 11 mm increase in rainfall.

Furthermore, we did not analyse the LT (heat-labile) and ST (heat-stabile) toxin expressions of the ETEC strains or various ETEC serotypes. Such analyses could have shed light on the seasonal variation found for ETEC, since diarrhoea is associated particularly strongly with ST-producing strains [36], and in the dry and rainy seasons different serotypes may predominate, some of them more diarrhoeagenic than others.

We did not have the opportunity to identify *Cryptosporidium* species at the genus level. It would have been particularly interesting to explore which species predominate during the rainy and dry seasons, as *C*. *hominis* has been reported to mostly account for waterborne outbreaks and *C*. *parvum* for foodborne outbreaks [57]. The seasonal patterns may have been affected by the fact that some pathogens, such as noroviruses, EAEC, *Salmonella* or *Campylobacter* may be detected in stools for some weeks after the resolution of clinical symptoms [10]. This should, however, mostly impact the cases found during the first weeks of the season.

Although the data does not provide information on actual incidences, the numbers of patients attending the health centre and agreeing to participate are given. It is possible that those with diarrhoea were more willing to participate, but this should not vary by season.

## Conclusion

Diarrhoeal pathogens are not equally presented over the year, but show seasonal variation with respect to precipitation in Guinea-Bissau. Waterborne pathogens such as *Cryptosporidium* can be expected during the rainy season, whereas viral pathogens appear more common during the dry season. Knowledge of pathogens’ seasonal variations could not only guide empiric treatment but also provide tools for devising interventions and preventive measures.

## Supporting information

S1 Fig The monthly proportion of diarrhoea and pathogens in respect to rainfall with a confidence interval; A) diarrhoea, B) any bacter, C) *Campylobacter*, D) EAEC, E) EIEC/*Shigella*, F) EPEC, G) ETEC, H) any parasite, I) *Cryptosporidium*, J) *D*. *fragilis*, K) *Giardia*, L) any virus, M) adenovirus 40/41, N) astrovirus, O) norovirus GI/GII, P) rotavirus A, Q) sapovirus. Data are not presented for EHEC (n = 6), *Salmonella* (n = 11), *V*. *cholerae* (n = 2), *Yersinia* (n = 3) and *E*. *histolytica* (n = 2).
(TIF)

S1 Data All relevant data for the study. (XLSX)

## References

[1] Levy K, Woster AP, Goldstein RS, Carlton EJ. Untangling the impacts of climate change on waterborne diseases: a systematic review of relationships between diarrheal diseases and temperature, rainfall, flooding, and drought. Environ Sci Technol. 2016 May 17;50(10):4905–22. doi: 10.1021/acs.est.5b06186 27058059 PMC5468171

[2] Lal A, Hales S, French N, Baker MG. Seasonality in human zoonotic enteric diseases: a systematic review. PloS One. 2012;7(4):e31883. doi: 10.1371/journal.pone.0031883 22485127 PMC3317665

[3] Lo Iacono G, Armstrong B, Fleming LE, Elson R, Kovats S, Vardoulakis S, et al. Challenges in developing methods for quantifying the effects of weather and climate on water-associated diseases: A systematic review. PLoS Negl Trop Dis. 2017 Jun;11(6):e0005659. doi: 10.1371/journal.pntd.0005659 28604791 PMC5481148

[4] Bissau Climate Weather Averages [Internet]. WorldWeatherOnline.com. [cited 2022 Aug 11]. https://www.worldweatheronline.com/bissau-weather/bissau/gw.aspx

[5] Kotloff KL, Blackwelder WC, Nasrin D, Nataro JP, Farag TH, van Eijk A, et al. The Global Enteric Multicenter Study (GEMS) of diarrheal disease in infants and young children in developing countries: epidemiologic and clinical methods of the case/control study. Clin Infect Dis Off Publ Infect Dis Soc Am. 2012 Dec;55 Suppl 4(Suppl 4):S232–245. doi: 10.1093/cid/cis753 23169936 PMC3502307

[6] Chao DL, Roose A, Roh M, Kotloff KL, Proctor JL. The seasonality of diarrheal pathogens: A retrospective study of seven sites over three years. PLoS Negl Trop Dis. 2019 Aug;13(8):e0007211. doi: 10.1371/journal.pntd.0007211 31415558 PMC6711541

[7] Perch M, Sodemann M, Jakobsen MS, Valentiner-Branth P, Steinsland H, Fischer TK, et al. Seven years’ experience with *Cryptosporidium parvum* in Guinea-Bissau, West Africa. Ann Trop Paediatr. 2001 Dec;21(4):313–8. 11732149 10.1080/07430170120093490

[8] Fischer TK, Valentiner-Branth P, Steinsland H, Perch M, Santos G, Aaby P, et al. Protective immunity after natural rotavirus infection: a community cohort study of newborn children in Guinea-Bissau, west Africa. J Infect Dis. 2002 Sep 1;186(5):593–7. doi: 10.1086/342294 12195345

[9] Fischer TK, Aaby P, Mølbak K, Rodrigues A. Rotavirus disease in Guinea-Bissau, West Africa: a review of longitudinal community and hospital studies. J Infect Dis. 2010 Sep 1;202 Suppl:S239–242. doi: 10.1086/653568 20684710

[10] Mero S, Timonen S, Lääveri T, Løfberg S, Kirveskari J, Ursing J, et al. Prevalence of diarrhoeal pathogens among children under five years of age with and without diarrhoea in Guinea-Bissau. PLoS Negl Trop Dis. 2021 Sep;15(9):e0009709. doi: 10.1371/journal.pntd.0009709 34587158 PMC8504977

[11] WHO. Diarrhoeal disease. [Internet]. [accessed 2023 Feb 13]. https://www.who.int/news-room/fact-sheets/detail/diarrhoeal-disease

[12] Antikainen J, Kantele A, Pakkanen SH, Lääveri T, Riutta J, Vaara M, et al. A quantitative polymerase chain reaction assay for rapid detection of 9 pathogens directly from stools of travelers with diarrhea. Clin Gastroenterol Hepatol Off Clin Pract J Am Gastroenterol Assoc. 2013;11(10):1300–1307.e3. doi: 10.1016/j.cgh.2013.03.037 23639597

[13] Greenland S, Drescher K. Maximum likelihood estimation of the attributable fraction from logistic models. Biometrics. 1993 Sep;49(3):865–72. 8241375

[14] Philipsborn R, Ahmed SM, Brosi BJ, Levy K. Climatic drivers of diarrheagenic *Escherichia coli* Incidence: a systematic review and meta-analysis. J Infect Dis. 2016 Jul 1;214(1):6–15. 26931446 10.1093/infdis/jiw081 PMC4907410

[15] Stoll BJ, Glass RI, Huq MI, Khan MU, Holt JE, Banu H. Surveillance of patients attending a diarrhoeal disease hospital in Bangladesh. Br Med J Clin Res Ed. 1982 Oct 23;285(6349):1185–8. doi: 10.1136/bmj.285.6349.1185 6812801 PMC1500105

[16] Guerrant RL, Kirchhoff LV, Shields DS, Nations MK, Leslie J, de Sousa MA, et al. Prospective study of diarrheal illnesses in northeastern Brazil: patterns of disease, nutritional impact, etiologies, and risk factors. J Infect Dis. 1983 Dec;148(6):986–97. doi: 10.1093/infdis/148.6.986 6361176

[17] Pai CH, Ahmed N, Lior H, Johnson WM, Sims HV, Woods DE. Epidemiology of sporadic diarrhea due to verocytotoxin-producing *Escherichia coli*: a two-year prospective study. J Infect Dis. 1988 May;157(5):1054–7. 3283256 10.1093/infdis/157.5.1054

[18] Colombatti R, Vieira CS, Bassani F, Cristofoli R, Coin A, Bertinato L, et al. Contamination of drinking water sources during the rainy season in an urban post-conflict community in Guinea Bissau: implications for sanitation priority. Afr J Med Med Sci. 2009 Jun;38(2):155–61. 20175419

[19] Alexander KA, Carzolio M, Goodin D, Vance E. Climate change is likely to worsen the public health threat of diarrheal disease in Botswana. Int J Environ Res Public Health. 2013 Mar 26;10(4):1202–30. doi: 10.3390/ijerph10041202 23531489 PMC3709313

[20] Horn LM, Hajat A, Sheppard L, Quinn C, Colborn J, Zermoglio MF, et al. Association between precipitation and diarrheal disease in Mozambique. Int J Environ Res Public Health. 2018 Apr 10;15(4). doi: 10.3390/ijerph15040709 29642611 PMC5923751

[21] Platts-Mills J, Houpt ER, Liu J, Zhang J, Guindo O, Sayinzoga-Makombe N, et al. Etiology and incidence of moderate-to-severe diarrhea in young children in Niger. J Pediatric Infect Dis Soc. 2021 Dec 31;10(12):1062–1070. doi: 10.1093/jpids/piab080 34468743 PMC8719619

[22] Bhavnani D, Goldstick JE, Cevallos W, Trueba G, Eisenberg JNS. Impact of rainfall on diarrheal disease risk associated with unimproved water and sanitation. Am J Trop Med Hyg. 2014 Apr;90(4):705–11. doi: 10.4269/ajtmh.13-0371 24567318 PMC3973516

[23] Jagai JS, Castronovo DA, Monchak J, Naumova EN. Seasonality of cryptosporidiosis: A meta-analysis approach. Environ Res. 2009 May;109(4):465–78. doi: 10.1016/j.envres.2009.02.008 19328462 PMC2732192

[24] Hunter PR, Thompson RCA. The zoonotic transmission of *Giardia* and *Cryptosporidium*. Int J Parasitol. 2005 Oct;35(11–12):1181–90. 16159658 10.1016/j.ijpara.2005.07.009

[25] Anyorikeya M, Ameme DK, Nyarko KM, Sackey SO, Afari E. Trends of diarrhoeal diseases in children under five years in the War Memorial Hospital-Navrongo, Ghana: 2010–2013. Pan Afr Med J. 2016;25(Suppl 1):8. doi: 10.11604/pamj.supp.2016.25.1.6173 28210376 PMC5292114

[26] Colston JM, Zaitchik BF, Badr HS, Burnett E, Ali S, Rayamajhi A, et al. Associations between eight earth observation—derived climate variables and enteropathogen infection: an independent participant data meta-analysis of surveillance studies with broad spectrum nucleic acid diagnostics. Geohealth. 2022 Jan 1;6(1). doi: 10.1029/2021GH000452 35024531 PMC8729196

[27] Alemayehu B, Ayele BT, Valsangiacomo C, Ambelu A. Spatiotemporal and hotspot detection of U5-children diarrhea in resource-limited areas of Ethiopia. Sci Rep. 2020 Jul 3;10(1):10997. doi: 10.1038/s41598-020-67623-0 32620796 PMC7335052

[28] Thiam S, Diène AN, Sy I, Winkler MS, Schindler C, Ndione JA, et al. Association between childhood diarrhoeal incidence and climatic factors in urban and rural settings in the health district of Mbour, Senegal. Int J Environ Res Public Health. 2017 Sep 12;14(9). doi: 10.3390/ijerph14091049 28895927 PMC5615586

[29] Darvesh N, Das JK, Vaivada T, Gaffey MF, Rasanathan K, Bhutta ZA. Water, sanitation and hygiene interventions for acute childhood diarrhea: a systematic review to provide estimates for the Lives Saved Tool. BMC Public Health. 2017 Nov 7;17(Suppl 4):776. doi: 10.1186/s12889-017-4746-1 29143638 PMC5688426

[30] WHO. Global progress report on WASH in health care facilities: Fundamentals first. 2021.

[31] Fayer R. *Cryptosporidium*: a water-borne zoonotic parasite. Vet Parasitol. 2004 Dec 9;126(1–2):37–56. 15567578 10.1016/j.vetpar.2004.09.004

[32] Lake IR, Bentham G, Kovats RS, Nichols GL. Effects of weather and river flow on cryptosporidiosis. J Water Health. 2005 Dec;3(4):469–74. doi: 10.2166/wh.2005.048 16459850

[33] Cacciò SM. Molecular epidemiology of *Dientamoeba fragilis*. Acta Trop. 2018 Aug;184:73–7. 28697994 10.1016/j.actatropica.2017.06.029

[34] Dixon BR. *Giardia duodenalis* in humans and animals—Transmission and disease. Res Vet Sci. 2021 Mar;135:283–9. 33066992 10.1016/j.rvsc.2020.09.034

[35] Steinsland H, Valentiner-Branth P, Perch M, Dias F, Fischer TK, Aaby P, et al. Enterotoxigenic *Escherichia coli* infections and diarrhea in a cohort of young children in Guinea-Bissau. J Infect Dis. 2002 Dec 15;186(12):1740–7. 12447759 10.1086/345817

[36] Platts-Mills JA, Babji S, Bodhidatta L, Gratz J, Haque R, Havt A, et al. Pathogen-specific burdens of community diarrhoea in developing countries: a multisite birth cohort study (MAL-ED). Lancet Glob Health. 2015;3(9):564. doi: 10.1016/S2214-109X(15)00151-5 26202075 PMC7328884

[37] Mason J, Iturriza-Gomara M, O’Brien SJ, Ngwira BM, Dove W, Maiden MCJ, et al. *Campylobacter* infection in children in Malawi is common and is frequently associated with enteric virus co-infections. PloS One. 2013;8(3):e59663. 23555739 10.1371/journal.pone.0059663 PMC3608717

[38] Mølbak K, Wested N, Højlyng N, Scheutz F, Gottschau A, Aaby P, et al. The etiology of early childhood diarrhea: a community study from Guinea-Bissau. J Infect Dis. 1994;169(3):581–7. doi: 10.1093/infdis/169.3.581 8158030

[39] Ouedraogo N, Ngangas SMT, Bonkoungou IJO, Tiendrebeogo AB, Traore KA, Sanou I, et al. Temporal distribution of gastroenteritis viruses in Ouagadougou, Burkina Faso: seasonality of rotavirus. BMC Public Health. 2017 Mar 21;17(1):274. doi: 10.1186/s12889-017-4161-7 28327111 PMC5359802

[40] Colston JM, Ahmed AM, Soofi SB, Svensen E, Haque R, Shrestha J, et al. Seasonality and within-subject clustering of rotavirus infections in an eight-site birth cohort study. Epidemiol Infect. 2018 Apr;146(6):688–697. doi: 10.1017/S0950268818000304 29534766 PMC9134355

[41] Patel MM, Pitzer VE, Alonso WJ, Vera D, Lopman B, Tate J, et al. Global seasonality of rotavirus disease. Pediatr Infect Dis J. 2013 Apr;32(4):e134–147. doi: 10.1097/INF.0b013e31827d3b68 23190782 PMC4103797

[42] Fan YM, Oikarinen S, Lehto KM, Nurminen N, Juuti R, Mangani C, et al. High prevalence of selected viruses and parasites and their predictors in Malawian children. Epidemiol Infect. 2019 Jan;147:e90. doi: 10.1017/S0950268819000025 30869004 PMC6521582

[43] Robilotti E, Deresinski S, Pinsky BA. Norovirus. Clin Microbiol Rev. 2015 Jan;28(1):134–64. doi: 10.1128/CMR.00075-14 25567225 PMC4284304

[44] Shamkhali Chenar S, Deng Z. Environmental indicators for human norovirus outbreaks. Int J Environ Health Res. 2017 Feb;27(1):40–51. doi: 10.1080/09603123.2016.1257705 27876423

[45] Bonkoungou IJ, Haukka K, Österblad M, Hakanen AJ, Traoré AS, Barro N, Siitonen A. Bacterial and viral etiology of childhood diarrhea in Ouagadougou, Burkina Faso. BMC Pediatr. 2013 Mar 19;13:36. doi: 10.1186/1471-2431-13-36 23506294 PMC3616825

[46] Addy PA, Antepim G, Frimpong EH. Prevalence of pathogenic *Escherichia coli* and parasites in infants with diarrhoea in Kumasi, Ghana. East Afr Med J. 2004 Jul;81(7):353–7. 15490707 10.4314/eamj.v81i7.9190

[47] Sambe-Ba B, Espié E, Faye ME, Timbiné LG, Sembene M, Gassama-Sow A. Community-acquired diarrhea among children and adults in urban settings in Senegal: clinical, epidemiological and microbiological aspects. BMC Infect Dis. 2013; 13: 580. doi: 10.1186/1471-2334-13-580 24321175 PMC3893462

[48] Lertsethtakarn P, Silapong S, Sakpaisal P, Serichantalergs O, Ruamsap N, Lurchachaiwong W, et al. Travelers’ diarrhea in Thailand: a quantitative analysis using TaqMan array card. Clin Infect Dis. 2018 Jun 18;67(1):120–127. 29351583 10.1093/cid/ciy040 PMC6248621

[49] Liu J, Platts-Mills JA, Juma J, Kabir F, Nkeze J, Okoi C et al. Use of quantitative molecular diagnostic methods to identify causes of diarrhoea in children: a reanalysis of the GEMS case-control study. Lancet. 2016 Sep 24;388(10051):1291–301. doi: 10.1016/S0140-6736(16)31529-X 27673470 PMC5471845

[50] Platts-Mills JA, Liu J, Rogawski ET, Kabir F, Lertsethtakarn P, Siguas M et al. Use of quantitative molecular diagnostic methods to assess the aetiology, burden, and clinical characteristics of diarrhoea in children in low-resource settings: a reanalysis of the MAL-ED cohort study. Lancet Glob Health 2018;6: e1309–18. doi: 10.1016/S2214-109X(18)30349-8 30287127 PMC6227251

[51] Lääveri T, Pakkanen SH, Antikainen J, Riutta J, Mero S, Kirveskari J et al. High number of diarrhoeal co-infections in travellers to Benin, West Africa. BMC Infect Dis. 2014 Feb 12;14:81. doi: 10.1186/1471-2334-14-81 24521079 PMC3928613

[52] Lääveri T, Vilkman K, Pakkanen SH, Kirveskari J, Kantele A. A prospective study of travellers’ diarrhoea: analysis of pathogen findings by destination in various (sub)tropical regions. Clin Microbiol Infect. 2018 Aug;24(8):908.e9–908.e16. doi: 10.1016/j.cmi.2017.10.034 29133155

[53] Li J, Wang Z, Karim MR, Zhang L. Detection of human intestinal protozoan parasites in vegetables and fruits: a review. Parasit Vectors. 2020 Jul 29;13(1):380. doi: 10.1186/s13071-020-04255-3 32727529 PMC7392835

[54] Ahmed D, Islam MS, Begum YA, Janzon A, Qadri F, Sjöling A. Presence of enterotoxigenic *Escherichia coli* in biofilms formed in water containers in poor households coincides with epidemic seasons in Dhaka. J Appl Microbiol. 2013 Apr;114(4):1223–9. 23279124 10.1111/jam.12109

[55] Adator EH, Cheng M, Holley R, McAllister T, Narvaez-Bravo C. Ability of Shiga toxigenic *Escherichia coli* to survive within dry-surface biofilms and transfer to fresh lettuce. Int J Food Microbiol. 2018 Mar 23;269:52–9. 29421358 10.1016/j.ijfoodmicro.2018.01.014

[56] Stolwijk AM, Straatman H, Zielhuis GA. Studying seasonality by using sine and cosine functions in regression analysis. J Epidemiol Community Health. 1999 Apr;53(4):235–8. doi: 10.1136/jech.53.4.235 10396550 PMC1756865

[57] Zahedi A, Ryan U. *Cryptosporidium*—an update with an emphasis on foodborne and waterborne transmission. Res Vet Sci. 2020 Oct;132:500–12. 32805698 10.1016/j.rvsc.2020.08.002

