# Supplementary figures and images for: Seasonal variation of diarrhoeal pathogens among Guinea-Bissauan children under five years of age
Source: PLoS Negl Trop Dis. 2023 Mar 13;17(3):e0011179. doi: 10.1371/journal.pntd.0011179 (PMC10035853; doi:10.1371/journal.pntd.0011179)

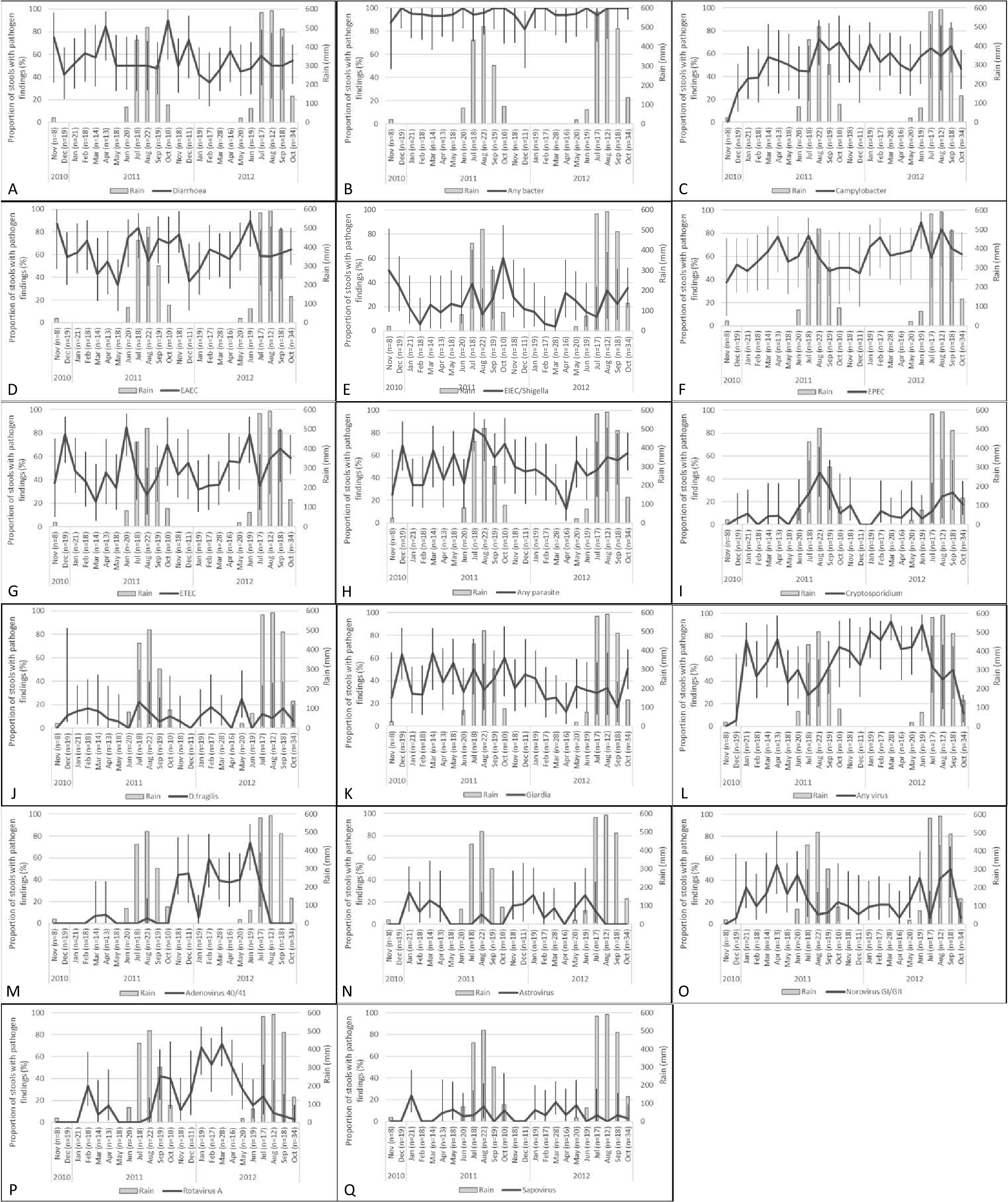

Supplement: S1 Fig — Data are not presented for EHEC (n = 6), Salmonella (n = 11), V. cholerae (n = 2), Yersinia (n = 3) and E. histolytica (n = 2). (TIF) [file pntd.0011179.s001.tif]
